# Notch and Fgf signaling during electrosensory versus mechanosensory lateral line organ development in a non-teleost ray-finned fish

**DOI:** 10.1016/j.ydbio.2017.08.017

**Published:** 2017-11-01

**Authors:** Melinda S. Modrell, Olivia R.A. Tidswell, Clare V.H. Baker

**Affiliations:** Department of Physiology, Development and Neuroscience, University of Cambridge, Anatomy Building, Downing Street, Cambridge CB2 3DY, UK

**Keywords:** Ampullary organs, Neuromasts, Electroreceptors, Hair cells, Fgf signaling, Notch signaling

## Abstract

The lateral line system is a useful model for studying the embryonic and evolutionary diversification of different organs and cell types. In jawed vertebrates, this ancestrally comprises lines of mechanosensory neuromasts over the head and trunk, flanked on the head by fields of electrosensory ampullary organs, all innervated by lateral line neurons in cranial lateral line ganglia. Both types of sense organs, and their afferent neurons, develop from cranial lateral line placodes. Current research primarily focuses on the posterior lateral line primordium in zebrafish, which migrates as a cell collective along the trunk; epithelial rosettes form in the trailing zone and are deposited as a line of neuromasts, within which hair cells and supporting cells differentiate. However, in at least some other teleosts (e.g. catfishes) and all non-teleosts, lines of cranial neuromasts are formed by placodes that elongate to form a sensory ridge, which subsequently fragments, with neuromasts differentiating in a line along the crest of the ridge. Furthermore, in many non-teleost species, electrosensory ampullary organs develop from the flanks of the sensory ridge. It is unknown to what extent the molecular mechanisms underlying neuromast formation from the zebrafish migrating posterior lateral line primordium are conserved with the as-yet unexplored molecular mechanisms underlying neuromast and ampullary organ formation from elongating lateral line placodes. Here, we report experiments in an electroreceptive non-teleost ray-finned fish, the Mississippi paddlefish *Polyodon spathula*, that suggest a conserved role for Notch signaling in regulating lateral line organ receptor cell number, but potentially divergent roles for the fibroblast growth factor signaling pathway, both between neuromasts and ampullary organs, and between paddlefish and zebrafish.

## Highlights

• Notch and Fgf pathway genes are expressed during paddlefish lateral line development.
• Fgf ligand genes are differentially expressed in neuromasts and ampullary organs.
• DAPT treatment results in irregular organ spacing and supernumerary receptor cells.
• SU5402 treatment yields fewer neuromasts, but ampullary organs form precociously.
• SU5402 treatment also results in supernumerary receptor cells.

## 1 Introduction

The mechanosensory lateral line system of fishes and aquatic amphibians comprises lines of neuromasts containing hair cells that are essentially identical to inner ear vestibular hair cells, and depolarise in response to local water movement (reviewed by Coombs et al., 1988; Webb, 2014). Neuromasts, and the afferent lateral line neurons that transmit information from neuromast hair cells to the hindbrain, develop from pre-otic and post-otic lateral line placodes, which elongate or migrate in characteristic lines over the head or trunk (reviewed by Gibbs, 2004; Webb, 2014; Piotrowski and Baker, 2014). Most current research into lateral line development uses as a model the zebrafish posterior lateral line placode, which migrates as a cell collective along the trunk, depositing a line of neuromasts in its wake (reviewed by Ghysen and Dambly-Chaudière, 2007; Aman and Piotrowski, 2011; Chitnis et al., 2012; Piotrowski and Baker, 2014; Thomas et al., 2015; Dalle Nogare and Chitnis, 2017). This also seems to be the mode of formation of cranial neuromast lines in zebrafish (see Piotrowski and Baker, 2014). However, in at least some teleosts (e.g. catfishes; Northcutt, 2003) and all non-teleosts, cranial neuromasts are formed by placodes that elongate to form a sensory ridge that subsequently fragments, with neuromasts differentiating in a line along the crest of the ridge (see Gibbs, 2004; Piotrowski and Baker, 2014).

Furthermore, in many non-teleosts, electrosensory ‘ampullary organs’ containing electroreceptor cells that depolarise in response to weak, low-frequency cathodal (exterior-negative) electric fields, differentiate on the flanks of some or all of the cranial sensory ridges formed by elongating lateral line placodes (reviewed by Gibbs, 2004; Baker et al., 2013). Experimental evidence for the lateral line placode origin of ampullary organs, as well as neuromasts, has now been provided for a lobe-finned bony tetrapod, the axolotl (Northcutt et al., 1995), a ray-finned non-teleost bony fish, the Mississippi paddlefish (Modrell et al., 2011a) and a cartilaginous fish, the little skate (Gillis et al., 2012). There is also substantial gene expression evidence for close developmental links between ampullary organs and neuromasts. Candidate gene approaches initially revealed that a handful of transcription factor and other genes, including the neurosensory microRNA *miR-183*, are expressed in ampullary organs as well as neuromasts, in axolotl (Metscher et al., 1997, Pierce et al., 2008, Modrell and Baker, 2012), paddlefish (Modrell et al., 2011b, Modrell et al., 2011a, Butts et al., 2014), shark and skate (Freitas et al., 2006, Gillis et al., 2012). More recently, we undertook an unbiased differential RNA-seq analysis in late-larval paddlefish, which revealed the expression in developing ampullary organs not only of multiple transcription factor genes required for hair cell development, including *Atoh1* (also reported in passing in Butts et al., 2014), but also of genes required for synaptic transmission at the hair cell ribbon synapse, including *Otoferlin*, which was previously thought to be unique to hair cells (Modrell et al., 2017). These data suggest close developmental, physiological and also evolutionary relationships between hair cells and non-teleost electroreceptors (Modrell et al., 2017).

Ampullary organs are lacking in frogs, as well as in neopterygian fishes, i.e., teleosts and their closest relatives (gars, bowfin), although anodally-sensitive ampullary-like organs have independently evolved at least twice in different teleost groups, most likely from neuromast hair cells (see Bullock et al., 1983; Alves-Gomes, 2001; Baker et al., 2013). The independent losses of ampullary organs in frogs and in neopterygian fishes (whether once in the neopterygian ancestor, or independently in the lineages leading to gars, bowfin and teleosts) may suggest that relatively simple genetic changes could result in the failure of lateral line placodes to form ampullary organs. It is unknown to what extent the little-explored molecular mechanisms underlying neuromast and ampullary organ formation from elongating lateral line placodes are conserved with those underlying neuromast formation from the well-studied migrating posterior lateral line primordium in the teleost zebrafish (Ghysen and Dambly-Chaudière, 2007, Aman and Piotrowski, 2011, Chitnis et al., 2012, Piotrowski and Baker, 2014, Thomas et al., 2015, Dalle Nogare and Chitnis, 2017).

Over the last decade, research from multiple labs has shown that the Fgf and Notch signaling pathways are critical for the formation of “protoneuromasts” - epithelial rosettes resulting from apical attachment and constriction of supporting cells around a central hair cell progenitor - within the trailing zone of the migrating zebrafish posterior lateral line primordium (reviewed by Aman and Piotrowski, 2011; Chitnis et al., 2012; Piotrowski and Baker, 2014; Thomas et al., 2015; Dalle Nogare and Chitnis, 2017). *Fgfr1* is expressed in the trailing zone of the migrating primordium, where it is activated by Fgf3 and Fgf10 from the leading zone, inducing the proneural transcription factor *Atoh1* (which is required for hair cell formation in both the inner ear and lateral line; Millimaki et al., 2007) and Notch ligand (*deltaA*) gene expression in the central hair cell progenitor (Millimaki et al., 2007, Aman and Piotrowski, 2008, Lecaudey et al., 2008, Nechiporuk and Raible, 2008). Atoh1 induces expression of another Notch ligand gene (*deltaD*) plus *Fgf10*, and inhibits expression of *Fgfr1*, so the central hair cell progenitor is a new focal source of Fgf10 and Notch ligands, which itself has low Fgf and Notch signaling (reviewed by Aman and Piotrowski, 2011; Chitnis et al., 2012; Piotrowski and Baker, 2014; Thomas et al., 2015). Fgf10 binds Fgfr1 in the surrounding cells, resulting in the maintenance of *Notch3* expression, which in turn responds to the Notch ligands from the hair cell progenitor by blocking *Atoh1* expression (preventing adoption of a hair cell fate). Fgf and Notch signaling pathway activity in the supporting cells also promotes apical constriction and cell adhesion, leading to epithelial rosette formation (Matsuda and Chitnis, 2010, Kozlovskaja-Gumbrienė et al., 2017).

Blocking Fgf signaling during zebrafish lateral line development prevents both *Atoh1* expression and epithelial rosette formation (Aman and Piotrowski, 2008, Lecaudey et al., 2008, Nechiporuk and Raible, 2008). Blocking Notch signaling, conversely, results in the progressive expansion of the expression of the key hair cell transcription factor gene *Atoh1*, which also results in the attenuation of Fgf signaling and failure of epithelial rosette maturation (Matsuda and Chitnis, 2010, Kozlovskaja-Gumbrienė et al., 2017).

To what extent are these mechanisms, which occur in the context of collective cell migration, conserved in neuromast formation, and indeed ampullary organ formation, within sensory ridges formed by elongating lateral line placodes? To our knowledge, only a single study has investigated the role of any signaling pathway in lateral line organ development in an electroreceptive species: retinoic acid treatment at late gastrula/early neurula stages in the axolotl led to the loss of most neuromasts and all ampullary organs, which was interpreted as a posteriorization effect (Gibbs and Northcutt, 2004). Here, we use as our model a chondrostean non-teleost ray finned fish, the Mississippi paddlefish *Polyodon spathula*, in which the large pre-otic lateral line placodes give rise to very large ampullary organ fields, as well as neuromast lines (Modrell et al., 2011a). We report the expression of selected members of the Notch and Fgf signaling pathways, and the effects of pathway inhibition using small molecule inhibitors, during lateral line organ development in this species. This revealed conservation of the role of Notch pathway activity in preventing adoption of a hair cell or electroreceptor fate in developing neuromasts and ampullary organs, respectively, but potentially significant differences in the likely roles of Fgf signaling, both between neuromasts and ampullary organs in paddlefish, and between paddlefish and zebrafish.

## 2 Materials and methods

### 2.1 Embryos

*Polyodon spathula* embryos were purchased from Osage Catfisheries Inc. (Osage Beach, MO, USA). Embryos were staged according to Bemis and Grande (1992). All experiments were performed in accordance with the approved institutional guidelines and regulations of the Institutional Animal Care and Use Committee of Kennesaw State University (approved protocol #12-001).

### 2.2 cDNA synthesis and cloning

Embryos were preserved in RNALater (Thermo Fisher Scientific, Inc., Waltham, MA, USA) overnight at 4 °C, then stored at −80 °C after removing excess solution. Total RNA from stage 40–46 embryos was isolated using Trizol (Thermo Fisher Scientific) and cDNA was synthesized using the Superscript III First Strand Synthesis kit (Thermo Fisher Scientific). cDNA fragments for probe synthesis were cloned using gene-specific primers, designed from paddlefish transcriptome sequences (Modrell et al., 2017). Standard PCR conditions were used to amplify cDNA fragments prior to cloning into the pDrive vector (Qiagen, Manchester, UK). Clones were sequenced from both strands (Department of Biochemistry Sequencing Facility, University of Cambridge, UK) and aligned using Sequencher (Gene Codes Corporation, Ann Arbor, MI, USA). Orthology was verified using NCBI's Basic Local Alignment Search Tool BLASTX (http://blast.ncbi.nlm.nih.gov/Blast.cgi). Sequences were deposited into GenBank with the following accession numbers: *fgf3* [MF185228], *fgf10* [MF185229], *fgf20* [MF185230], *fgfr1* [MF185231], *hes-5-like* [MF185232], *jagged1* [MF185233], *Notch*1 [MF185234].

### 2.3 In situ hybridization

Whole-mount in situ hybridization was performed as described (Modrell et al., 2011a). Anti-sense RNA probes were synthesized using T7 or SP6 polymerases (Promega, Southhampton, UK) and digoxigenin-labeled dUTPs (Roche, Basal, Switerland).

### 2.4 Drug treatments and immunohistochemistry

Embryos were soaked for 18–24 h at 18 °C in a Petri dish containing water with 50 µM or 100 µM of SU5402 (Tocris Bioscience, Bristol, UK) or DAPT (Sigma-Aldrich) (stock solutions were diluted in dimethyl sulfoxide [DMSO] and frozen prior to use), or 1–2% DMSO (control). For each condition, at least two trials were performed, each starting with at least 15 individuals (of different clutches). After treatment, embryos were transferred to new dishes and rinsed thoroughly 3 or 4 times in water. Some embryos were fixed immediately post-treatment in modified Carnoy's fixative (6 volumes ethanol: 3 volumes 37% formaldehyde: 1 volume glacial acetic acid). The remaining embryos were allowed to develop to desired stages prior to fixation. All fixed embryos were dehydrated into absolute ethanol for storage, prior to rehydration for whole-mount immunostaining, which was performed as described (Modrell et al., 2011a) using anti-bullfrog Parvalbumin-3 (Heller et al., 2002) (rabbit IgG, 1:15,000–30,000; a kind gift from A. J. Hudspeth, Rockefeller University, NY, USA), which labels both hair cells and electroreceptors (Modrell et al., 2011a). The secondary antibody was horseradish peroxidase-conjugated goat anti-rabbit (1:600; Jackson ImmunoResearch Laboratories, West Grove, PA, USA).

## 3 Results

We aimed to assess the extent to which the roles of Notch and Fgf signaling in neuromast formation from the migrating zebrafish posterior lateral line primordium are likely to be conserved in the development of neuromasts and/or ampullary organs from elongating lateral line placodes in a non-teleost ray-finned fish, the Mississippi paddlefish *Polyodon spathula*. In this species, the three post-otic lateral line placodes (middle, supratemporal and posterior) only give rise to neuromasts, but the three pre-otic lateral line placodes (anterodorsal, anteroventral and otic) give rise to both neuromasts and ampullary organs, with particularly large ampullary organ fields arising from the very large anterodorsal and anteroventral lateral line placodes (Modrell et al., 2011a). Ampullary organs differentiate significantly later than neuromasts (Modrell et al., 2011a): Fig. 1 shows a schematic time-line for key stages of paddlefish development, with an emphasis on lateral line development (Bemis and Grande, 1992, Modrell et al., 2011a).

**Fig. 1:**
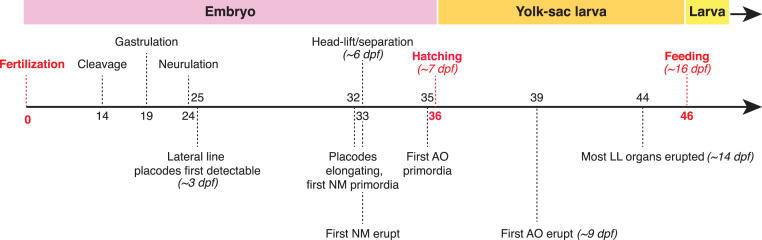
Timeline for paddlefish development, with an emphasis on lateral line development. Events defining boundaries between stages of development (embryo, yolk-sac larva and feeding larva) are marked in red, with approximate timings (days post fertilization [dpf]) given for development at 18 °C. Adapted from Bemis and Grande (1992) and Modrell et al. (2011a). Abbreviations: **AO**, ampullary organs; **dpf**, days post-fertilization; **LL**, lateral line; **NM**, neuromasts.

### 3.1 Notch signaling pathway genes are expressed during paddlefish lateral line development

In order to investigate the likely role of Notch signaling during cranial lateral line placode development in paddlefish, we cloned the receptor gene *Notch1*, the ligand gene *Jagged1 (Jag1)*, and a presumed Notch effector gene that was most closely related to *Hes5* (*Hes5-like*), and examined their expression by whole-mount in situ hybridization.

**Fig. 2:**
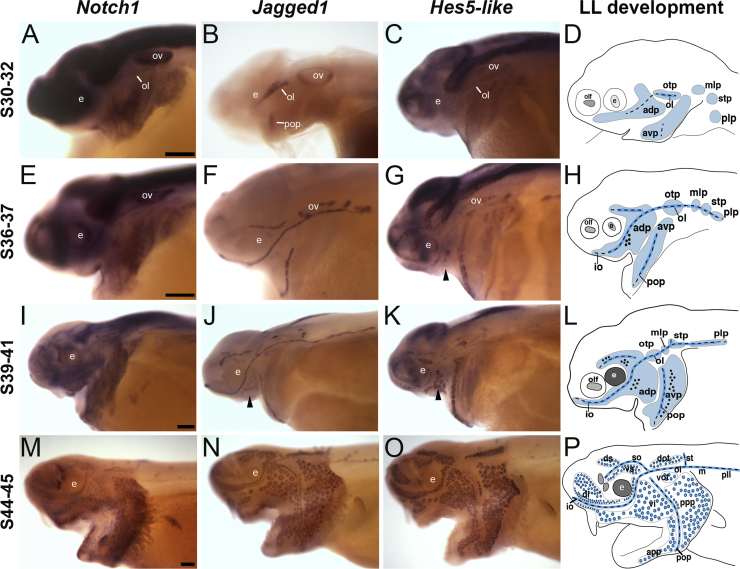
Notch signaling pathway genes are expressed in the developing lateral line system in paddlefish. Whole-mount in situ hybridization for paddlefish *Notch1*, *Jag1* and *Hes-5-like* at different stages, with schematic representations of lateral line development, modified from Modrell et al. (2011a), showing lateral line placode/organ development at different stages in shades of blue, elongating placodes in light blue and emerging neuromast canal lines/ampullary organs in darker blue. Notch pathway gene expression is indicated within those tissues or organs, depending on stage, in black. (A-D) At stages 30–32, *Notch1*, *Jag1* and *Hes5-like* all seem to be expressed in the developing otic neuromast line between the eye and otic vesicle; *Notch1* and *Hes5-like* are strongly expressed in the brain, eye and otic vesicle, and in the region of the pre-otic lateral line placodes. *Jag1* expression is restricted to the developing otic neuromast line and prospective opercular line, plus the otic vesicle. (E-H) At stages 36–37, the lateral line expression of these Notch pathway transcripts includes the other pre-otic neuromast lines and post-otic lateral line primordia. Weak, patchy expression of *Jag1* ventral to the pre-otic neuromast lines likely represents developing ampullary organ fields (F). Similarly, *Hes5-like* expression is observed in the developing ventral infraorbital ampullary organ field (arrowhead in G). (I-L) At stages 39–41, although lateral line expression of *Notch1* (I) is difficult to observe, expression of *Jag1* (J) and *Hes5-like* (K) is present in all neuromast lines, including the posterior lateral line, while expression of *Jag1* and *Hes5-like* is also seen in the flanking ampullary organ fields (J,K: arrowheads indicate the ventral infraorbital ampullary organ field). (M-P) At stages 44–45, expression of *Notch1* (J), *Jag1* (K) and *Hes5-like* (L) is seen in both neuromasts and ampullary organs. Abbreviations: **adp**, anterodorsal lateral line placode; **app**, anterior preopercular ampullary field; **avp**, anteroventral lateral line placode; **dot**, dorsal otic ampullary field; **di**, dorsal infraorbital ampullary field; **ds**, dorsal supraorbital ampullary field; **e**, eye; **epi**, epibranchial placode region; **io**, infraorbital lateral line; **LL**, lateral line; **m**, middle lateral line; **mlp**, middle lateral line placode; **ol**, otic lateral line; **olf**, olfactory; **otp**, otic lateral line placode; **ov**, otic vesicle; **pll**, posterior lateral line; **plp**, posterior lateral line placode; **pop**, preopercular lateral line; **ppp**, posterior preopercular ampullary field; **S**, stage; **so**, supraorbital lateral line; **st**, supratemporal lateral line; **stp**, supratemporal lateral line placode; **vi**, ventral infraorbital ampullary field; **vot**, ventral otic ampullary field; **vs**, ventral supraorbital ampullary field. Scale bars: 200 µm.

Lateral line expression of *Notch1*, *Jag1* and *Hes5-like* was first seen around stages 30–32 (encompassing 4–5 h of development at 18 °C; Fig. 1; Fig. 2A–D), when the pre-otic placodes have already started elongating over the head, and the first neuromast primordia can be detected by histology in the otic line, between the otic vesicle and the eye, although they have not yet emerged above the epidermal layer (Modrell et al., 2011a). This was clearest for *Jag1*, whose expression was restricted to the developing otic neuromast line, the prospective preopercular neuromast line on the gill flap, and the otic vesicle (Fig. 2B). The broader expression patterns seen for *Notch1* and *Hes5-like*, with particularly strong expression in the brain, eyes and otic vesicle, made their lateral line-specific expression harder to discern, although both at least seemed to be expressed in the developing otic neuromast line (Fig. 2A,C).

By stages 36–37, when the first functional neuromasts (as determined by uptake of the styryl dye FM1-43) are present in the otic line, and the first ampullary organ primordia are already detectable by histology (Modrell et al., 2011a), expression of all three Notch pathway genes was seen in the pre-otic neuromast lines, and in the posterior lateral line primordium (Fig. 2E–H). Again, this was most obvious for *Jag1*, whose expression was restricted to the developing lateral line system, now including the post-otic lateral line primordia, and the otic vesicle (Fig. 2F). *Jag1* expression was seen in the pre-otic neuromast lines, with some weak, patchy expression ventral to the supraorbital, infraorbital and preopercular neuromast lines that likely represents developing ampullary organ fields (Fig. 2F). *Notch1* expression was harder to ascertain, given its broader expression in this region (Fig. 2E). However, weak *Hes5-like* expression was observed in the developing ventral infraorbital ampullary organ field (arrowhead, Fig. 2G), as well as in the pre-otic neuromast lines and post-otic lateral line primordia (Fig. 2G).

By stages 39–41, when ampullary organs are erupting to the surface (Modrell et al., 2011a), the expression of *Notch1*, *Jag1* and *Hes5-like* was maintained in the pre-otic and post-otic neuromast lines, and was expanding in the ventral infraorbital ampullary organ field and turning on in other fields (Fig. 2I–L), although this was difficult to see for *Notch1*, mainly due to underlying widespread expression (Fig. 2I).

By stages 44–45, when almost all ampullary organs are fully differentiated (Modrell et al., 2011a), expression of *Notch1*, *Jag1* and *Hes5-like* was clearly evident in all neuromast lines and ampullary organ fields (Fig. 2M–P).

Overall, the continual expression of Notch pathway genes throughout paddlefish lateral line placode development is consistent with an important role for the Notch pathway in neuromast and ampullary organ formation from elongating lateral line placodes.

### 3.2 Notch inhibition results in irregularly spaced lateral line organs with supernumerary hair cells/electroreceptors

**Fig. 3:**
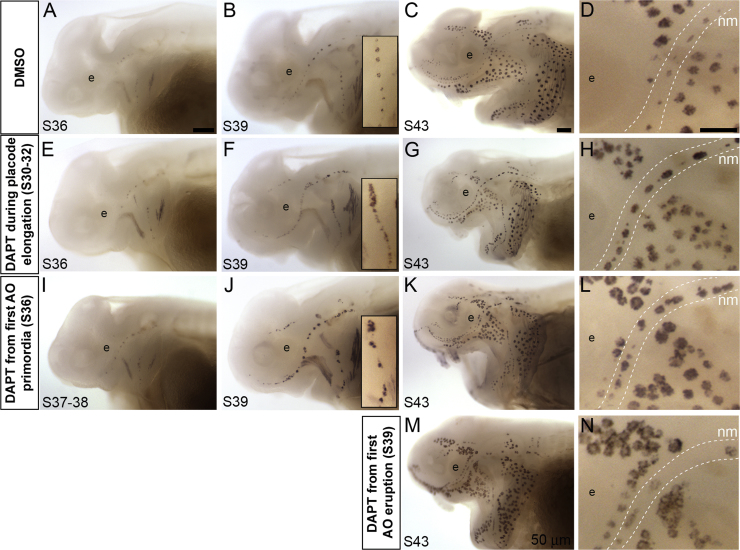
DAPT treatment during lateral line development in paddlefish results in irregularly spaced sensory organs with supernumerary receptor cells. Whole-mount immunostaining using an antibody raised against bullfrog parvalbumin-3 (Heller et al., 2002), which labels paddlefish hair cells and electroreceptors (Modrell et al., 2011a). (A-D) DMSO control embryos at stages 36 (A), stage 39 (B; inset shows higher power view of preopercular neuromast line) and stage 43 (C,D), for stage-matched comparison with drug-treated embryos. Panel D shows a higher-power view of the region caudal to the eye from the embryo in C, showing the infraorbital neuromast line and the flanking ampullary organ fields. Dotted lines indicate approximate boundaries of the neuromast line. (E-H) Embryos treated with 50 μM or 100 μM DAPT for 18–24 h during placode elongation (stages 30–32), analyzed at stage 36 (i.e., immediately post-treatment; E), stage 39 (F; inset shows higher power view of preopercular neuromast line) and stage 43 (G,H). By stage 39 onwards, neuromast lines contain more neuromasts, irregularly spaced and with more hair cells, than seen in stage-matched controls. (I-L) Embryos treated with 50 μM or 100 μM DAPT for 18–24 h from stage 36, when the first ampullary organ primordia are already forming, analyzed immediately post-treatment at stages 37–38 (I), and at stage 39 (J; inset shows higher power view of preopercular neuromast line) and stage 43 (K,L). From stage 39 onwards, embryos have more neuromasts, irregularly spaced and with more hair cells. At stage 43, ampullary organs contain more electroreceptors and are clustered together in places. (M,N) Embryo treated with 50 μM DAPT for 18–24 h from stage 39, when ampullary organs start to erupt, and analyzed at stage 43. Neuromasts contain more hair cells and are irregularly spaced; ampullary organs contain more electroreceptors and are clustered together, forming large patches. Abbreviations: **ao**, ampullary organs; **e**, eye; **nm**, neuromasts; **S**, stage. Scale bars: 200 µm except for D,H,L,N, 100 µm.

In order to provide an initial assessment of the role of Notch signaling during lateral line sensory organ development in paddlefish, we treated embryos with the γ-secretase inhibitor DAPT (either 50 or 100 μM) for 18–24 h at different stages of lateral line development. Drug-treated embryos were analyzed by whole-mount immunostaining using an antibody raised against bullfrog parvalbumin3 (Heller et al., 2002), which labels hair cells and electroreceptors (Modrell et al., 2011a). We did not see any dose-dependent effects, so we have combined the numbers for embryos treated with 50 or 100 μM DAPT: these exhibited the same lateral line phenotypes, as well as being developmentally delayed and displaying gross morphological defects characteristic of Notch pathway inhibition (e.g. abnormal tailbuds, cranial edema). Fig. 3A–D shows stage-matched DMSO controls for the stages at which DAPT-treated embryos were analyzed, to account for any developmental delays (stage 36, n = 7; stage 39, n = 10; stage 43, n = 16).

When embryos were treated with DAPT for 18–24 h from stages 30–32 (i.e., from pre-otic lateral line placode elongation stages, just before the first neuromast organ primordia form), no effect was seen immediately post-treatment at stage 36 (n = 7/7; Fig. 3E). At stage 39, DMSO control embryos exhibited well defined lines of neuromasts, each consisting of a few hair cells (n = 10/10; Fig. 3B). In contrast, in DAPT-treated stage 39 embryos, individual neuromasts within lines were poorly defined, often clustering together, with many hair cells (n = 13/13; Fig. 3F). At stage 43, in DMSO control embryos, both neuromast lines and ampullary organ fields were present and the sensory organs were well defined and regularly spaced (n = 16/16; Fig. 3C,D). In DAPT-treated embryos, neuromasts were more clearly defined than at stage 39, but more numerous, with many more hair cells, and irregularly spaced (n = 26/26; Fig. 3G,H). Ampullary organs, in contrast, appeared largely unaffected (Fig. 3G,H). These data suggest that Notch signaling during placode elongation stages is important for regulating neuromast spacing and hair cell number, but not for ampullary organ spacing or electroreceptor development.

DAPT treatment for 18–24 h from stage 36, when the first functional neuromasts are present in the otic line and the first ampullary organ primordia are already detectable by histology (Modrell et al., 2011a), similarly resulted in embryos with more hair cells in neuromasts, except in embryos immediately post-treatment at stages 37–38, in which neuromasts appeared normal (n = 4/4; Fig. 3I). By stage 39, neuromasts contained more hair cells per organ in DAPT-treated embryos than in stage-matched DMSO controls (n = 5/5; Fig. 3J; compare with Fig. 3B). The neuromasts themselves were generally more clearly separated than was seen after DAPT treatment from stages 30–32 (compare Fig. 3J with Fig. 3F), but they were still irregularly spaced. By stage 43, neuromasts still contained more hair cells and exhibited irregular spacing, while ampullary organs also contained more electroreceptors and were clustered together in places (n = 9/9; Fig. 3K,L; compare with Fig. 3C,D). Taken together, these data suggest that Notch signaling at this later stage, during the development of ampullary organ primordia, is still required to regulate neuromast spacing and the number of hair cells per organ, and is also required to regulate ampullary organ spacing and the number of electroreceptors within each organ.

To determine if Notch signaling continues to affect hair cell and electroreceptor differentiation at even later stages, after ampullary organs have started to erupt to the surface, we treated embryos with 50 μM DAPT at stage 39 for 18–24 h, and analyzed them around stage 43. As was seen after DAPT treatment from stage 36, neuromasts had more hair cells and were irregularly spaced, and ampullary organs seemed to have more electroreceptors and were clustered together (n = 10/10; Fig. 3M,N), in some areas forming large patches of contiguous ampullary organs (Fig. 3N). These effects of Notch inhibition at ampullary organ eruption stages suggest that persistent Notch signaling is required during lateral line development in paddlefish, both to regulate the numbers of hair cells and electroreceptors per organ and also the usual spacing of neuromasts and ampullary organs.

### 3.3 Paddlefish *fgfr1* and the ligand genes *fgf3* and *fgf20* are expressed during both neuromast and ampullary organ development, but *fgf10* is restricted to the mechanosensory system

**Fig. 4:**
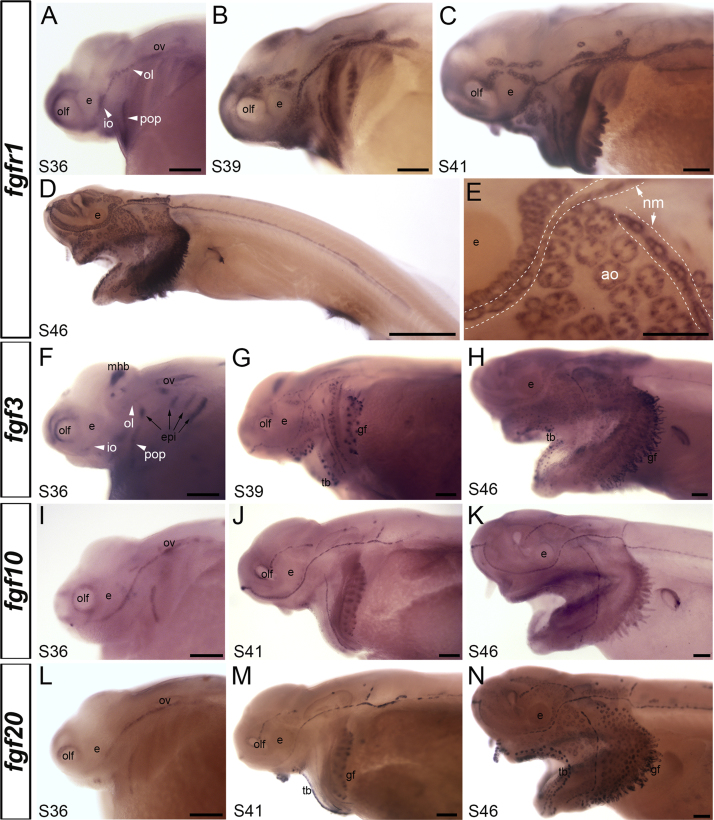
Fgf signaling pathway gene expression during lateral line organ development in paddlefish. Whole-mount in situ hybridization in paddlefish embryos for the indicated genes and stages. (A-E) *Fgfr1* is expressed in the otic, infraorbital and preopercular neuromast lines at stage 36 (A) and at stage 39 (B), expression is also seen in the developing ampullary organ fields flanking the neuromast lines. At both stage 41 (C) and stage 46 (D,E), *fgfr1* continues to be expressed in neuromast lines and ampullary organ fields. Panel E shows a higher-power view of the area caudal to the eye at stage 46: dotted lines indicate approximate boundaries of the neuromast lines. (F-H) *Fgf3* expression at stage 36 (F) is strong in the midbrain-hindbrain boundary, olfactory system and epibranchial placodes, and weak in the infraorbital, otic and preopercular neuromast lines. By stage 39 (G), *fgf3* is more strongly expressed in neuromast lines and ampullary organ fields; expression is also seen in gill filaments and taste buds. This expression pattern persists through stage 46 (H). (I-K) *Fgf10* expression is seen at stage 36 (I) in the otic, infraorbital and preopercular neuromast lines, and in all neuromast lines at stage 41 (J) and stage 46 (K). *Fgf10* is not expressed in the ampullary organ fields at any stage. (L-N) *Fgf20* is expressed in the otic, infraorbital and preopercular neuromast lines at stage 36 (L) and in all neuromast lines at stage 41 (M), when it is also expressed in taste buds and gill filaments. At stage 46 (N), *fgf20* continues to be expressed in all neuromast lines and is now also expressed in ampullary organs. Abbreviations: **ao**, ampullary organ; **e**, eye; **epi**, epibranchial placodes, **gf**, gill filaments; **io**, infraorbital lateral line; **mhb**, midbrain-hindbrain boundary; **nm**, neuromast; **ol**, otic lateral line; **olf**, olfactory epithelium; **ov**, otic vesicle; **pop**, preopercular neuromast line; **S**, stage; **tb**, taste buds. Scale bars: 200 µm except D, 1 mm.

In zebrafish, Fgfr1 in the trailing zone of the migrating posterior lateral line primordium is activated by Fgf3 and Fgf10 from the leading zone, inducing *Atoh1* and Notch ligand (*deltaA*) gene expression in the central hair cell progenitor (Millimaki et al., 2007, Aman and Piotrowski, 2008, Lecaudey et al., 2008, Nechiporuk and Raible, 2008). At stage 36 in paddlefish, when the first functional neuromasts are present in the otic line and the first ampullary organ primordia can already be detected by histology (Modrell et al., 2011a), *fgfr1* expression was observed in the otic, infraorbital and preopercular neuromast lines (Fig. 4A). By stage 39, when ampullary organs first start to erupt to the surface (Modrell et al., 2011a), *fgfr1* transcripts were also evident in the developing ampullary organ fields that flank the neuromast lines (Fig. 4B). *Fgfr1* expression was maintained in all neuromast lines and ampullary organ fields at stages 41 and 46 (Fig. 4C–E).

As regards ligand genes, paddlefish *fgf3* expression was weakly seen at stage 36 in the otic, infraorbital and preopercular neuromast lines, with stronger expression in other tissues such as the epibranchial placodes and midbrain-hindbrain boundary (Fig. 4F). By stage 39, *fgf3* was expressed in both neuromast lines and developing ampullary organ fields, as well as in taste buds and gill filaments (Fig. 4G). This expression pattern persisted through stage 46 (Fig. 4H).

At stage 36, paddlefish *fgf10* was strongly expressed in the otic, infraorbital and preopercular neuromast lines (Fig. 4I). At stage 41, *fgf10* expression continued in all the neuromast lines, but was absent from the developing ampullary organ fields (Fig. 4J). This pattern was maintained through at least stage 46 (Fig. 4K), when ampullary organs are fully differentiated (Modrell et al., 2011a), suggesting that *fgf10* is neuromast-specific.

Finally, we examined the expression of *fgf20*, which has not been reported in the zebrafish lateral line system, to our knowledge, but which is important for the development of a subset of outer hair cells in the mouse cochlea (Hayashi et al., 2008, Huh et al., 2012, Huh et al., 2015). Like *fgfr1* and *fgf3*, paddlefish *fgf20* was expressed at stage 36 in the otic, infraorbital and preopercular neuromast lines (Fig. 4L). By stage 41, *fgf20* expression was seen in all the neuromast lines (as well as in taste buds and gill filaments), but not in the ampullary organ fields (Fig. 4M). By stage 46, *fgf20* was expressed in ampullary organs, though more weakly than in the neuromast lines (Fig. 4N). Although the precise stage at which *fgf20* transcripts are first detectable in ampullary organs was not determined, it is certainly well after stage 39, when the ampullary organs have begun to erupt to the surface (Modrell et al., 2011a) and express *fgfr1* and *fgf3* (Fig. 4B,G).

### 3.4 Expression of Fgf pathway genes in mature lateral line organs reveals spatially restricted domains of expression

**Fig. 5:**
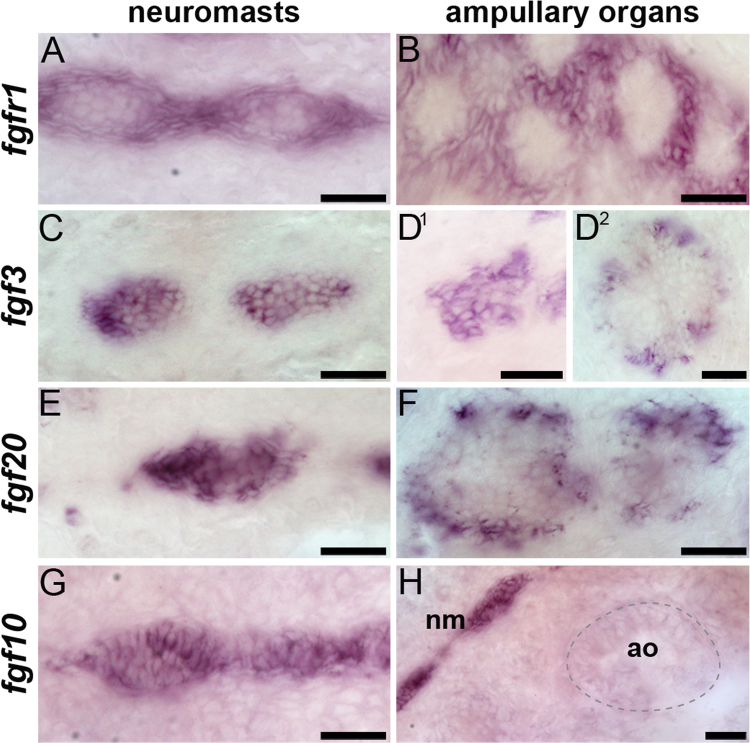
Differential expression of Fgf pathway genes in mature paddlefish lateral line organs. Skin-mount preparations from the same infraorbital region of stage 46 paddlefish embryos, following whole-mount in situ hybridization for the indicated genes. (A,B) *Fgfr1* is expressed more strongly at the periphery of neuromasts and in interneuromast cells than in the central neuromast domain (A), and is excluded from the central domain of ampullary organs (B). (C-D^2^) *Fgf3* is centrally expressed in neuromasts (C) and in smaller (presumably younger) ampullary organs (D^1^). However, in larger (presumably mature) ampullary organs, *fgf3* is expressed more strongly, and patchily, in a subset of peripheral cells (D^2^). (E,F) *Fgf20* is expressed in the central domain of neuromasts (E), but in ampullary organs, it is expressed much more strongly, and patchily, in a subset of peripheral cells (F). (G,H) *Fgf10* is expressed throughout neuromasts and in interneuromast cells (G,H), but not in ampullary organs (H). The dotted line in panel H outlines the approximate boundary of an ampullary organ. Abbreviations: **ao**, ampullary organ; **nm**, neuromasts. Scale bar: 20 µm.

Closer examination of the expression patterns of the four Fgf signaling pathway genes analyzed at stage 46, when cranial neuromasts and ampullary organs are fully differentiated (Modrell et al., 2011a), revealed differences in expression domains within and between lateral line organ types. Paddlefish *fgfr1* appeared to be strongly expressed at the periphery of neuromasts and within the cells connecting adjacent neuromasts, and only weakly in the hair cell-containing sensory epithelium (Fig. 5A). Similarly, in the ampullary organs, *fgfr1* was excluded from the centrally located sensory cell-containing domain but was strongly expressed in the peripheral part of the organ (and adjacent ectodermal cells) (Fig. 5B). In contrast, expression of the ligand gene *fgf3* was centrally localized in neuromasts and in smaller (presumably younger) ampullary organs (Fig. 5C,D^1^). In larger (presumably more mature) ampullary organs, although weak expression was still observed throughout the sensory epithelium, *fgf3* appeared to be expressed more strongly by a subset of peripheral cells (Fig. 5D^2^). Similarly, expression of the ligand gene *fgf20* was restricted to the central hair cell-containing domain of neuromasts (Fig. 5E), while in ampullary organs, *fgf20* was peripherally localized, with a subset of cells showing particularly strong expression (Fig. 5F). In contrast to *fgf3* and *fgf20*, the ligand gene *fgf10* was expressed in both central and peripheral domains of the neuromasts and in interneuromast cells (Fig. 5G,H), with no expression in ampullary organs (Fig. 5H).

Overall, our expression analysis has revealed that *fgfr1* and *fgf* ligand genes are expressed in developing ampullary organs, as well as neuromasts. However, the restriction of *fgf10* to the mechanosensory system, the significantly later expression of *fgf20* than *fgf3* in developing ampullary organs, and the differing spatial localization of all three ligand genes within mature organs, suggest that the roles of Fgf signaling via these ligands are likely to differ both within and between different lateral line organ types.

### 3.5 Fgf inhibition during different stages of sensory organ development causes patterning defects in both neuromasts and ampullary organs

In order to gain insight into the potential roles of Fgf signaling in the development of cranial neuromasts and ampullary organs from elongating lateral line placodes in paddlefish, we treated embryos at different stages of lateral line development with the broad spectrum Fgf receptor inhibitor SU5402 (50 or 100 μM) or DMSO as a control. Embryos were fixed at various developmental stages post-treatment and immunostained for hair cells and electroreceptors using the anti-bullfrog parvalbumin3 antibody (Heller et al., 2002, Modrell et al., 2011a). At both concentrations of SU5402, we observed highly curved tails, which is characteristic of blocking Fgf signaling (e.g. Draper et al., 2003; Griffin and Kimelman, 2003). Both concentrations also resulted in developmental delays, so SU5402-treated embryos were compared with stage-matched DMSO controls. No lateral line-specific phenotypes were seen after 50 μM SU5402 treatment at any stage.

**Fig. 6:**
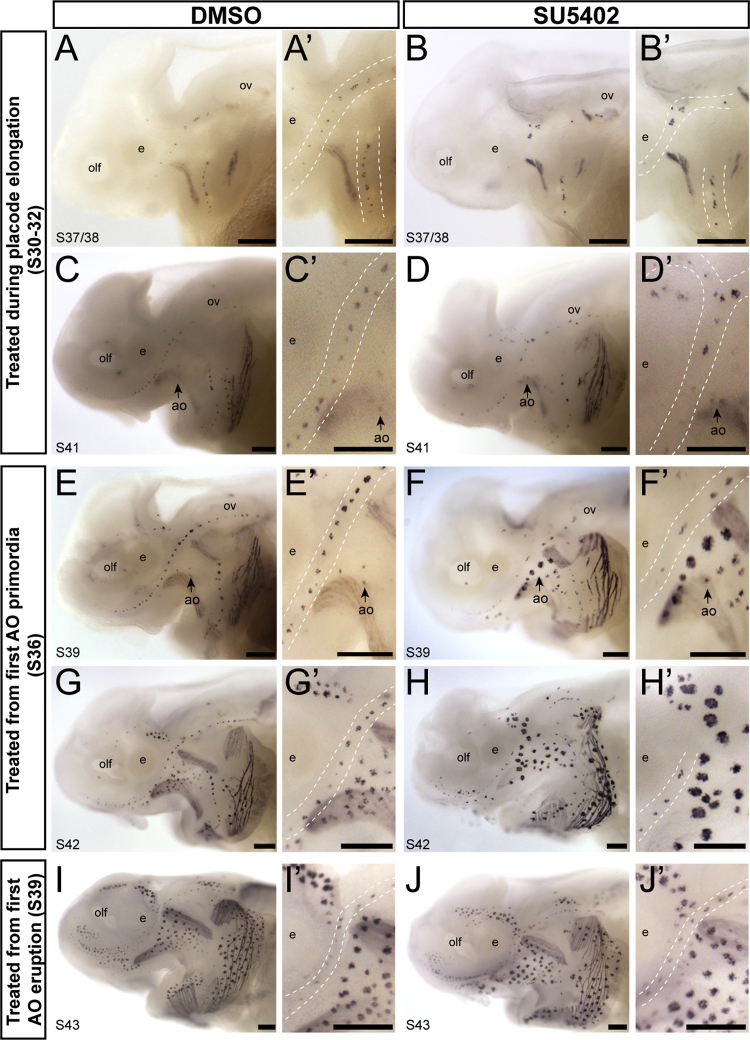
SU5402 treatment during paddlefish lateral line organ development yields contrasting phenotypes in neuromasts and ampullary organs. Whole-mount immunostaining using an antibody raised against bullfrog parvalbumin-3 (Heller et al., 2002), which labels paddlefish hair cells and electroreceptors (Modrell et al., 2011a). Dotted lines in higher-power views indicate approximate boundaries of neuromast lines. (A-D^’^) Embryos treated for 18–24 h from stages 30–32 (placode elongation stages, with the first neuromast primordia detectable at stage 32). At both stage 37/8 (A-B′) and stage 41 (C-D′), comparison of 100 μM SU5402-treated embryos with stage-matched DMSO controls reveals fewer neuromasts, some with more hair cells, though no obvious effect on ampullary organs. (E-H′) Embryos treated for 18–24 h from stage 36, when the first ampullary organ primordia are already detectable by histology. At stage 39, when ampullary organs begin to erupt (E-F′), comparison of 100 μM SU5402-treated embryos with stage-matched DMSO controls reveals fewer neuromasts, though with no obvious effect on hair cell number, and the precocious emergence of ampullary organs, some with many more electroreceptors than seen in DMSO controls. At stage 42 (G-H′), fewer neuromasts were seen in 100 μM SU5402-treated embryos than in stage-matched DMSO controls, again with no obvious change in hair cell number, while ampullary organs were present in normal numbers but with more electroreceptors. (I-J′) Embryos treated for 18–24 h from stage 39, when ampullary organs start to erupt. At stage 43, comparison of 50 μM SU5402-treated embryos with stage-matched DMSO controls showed no effect on lateral line organs. Abbreviations: **ao**, ampullary organ; **e**, eye; **olf**, olfactory system; ov, otic vesicle; **S**, stage. Scale bars: 200 µm.

Compared to DMSO control embryos (n = 10; Fig. 6A,A′), Fgf inhibition for 18–24 h during placode elongation (stages 30–32) resulted in fewer neuromasts by stage 37/38, but those that were present appeared to have more hair cells (n = 4/4; Fig. 6B,B′). By stage 41, DMSO control embryos had many neuromasts, and some ampullary organs were also present (n = 6; Fig. 6C,C′), while 100 μM SU5402-treated embryos continued to have fewer neuromasts, some with many more hair cells than seen in DMSO controls (n = 7/8; Fig. 6D,D′). As was the case for DAPT treatment, there was no effect on ampullary organ formation of SU5402 treatment at placode elongation stages.

In contrast, 100 μM SU5402 treatment for 18–24 h from stage 36, when the first ampullary organ primordia are already forming, affected both neuromasts and ampullary organs, though in a contrasting manner (Fig. 6E-H′). In DMSO control embryos analyzed at stage 39 (when ampullary organs begin to erupt), many neuromasts were present, but very few electroreceptors were observed (n = 10; Fig. 6E,E′). In 100 μM SU5402-treated embryos of the equivalent stage, fewer neuromasts were seen, though the effect was not as severe as following treatment at stages 30–32, and hair cell number appeared largely unaffected (Fig. 6D,D′). However, many precocious ampullary organs were present, some consisting of many more electroreceptors than seen in DMSO controls (n = 12/13; Fig. 6F,F′). By stage 42, fewer neuromasts were still observed in 100 μM SU5402-treated embryos, with no obvious effect on the number of ampullary organs, although they consisted of many more electroreceptors (DMSO control n = 7; Fig. 6G,G′; SU5402-treated n = 4/4; Fig. 6H,H′). These results suggest that Fgf inhibition at stage 36, when ampullary organ primordia are forming, has opposing effects on neuromasts versus ampullary organs.

We also treated embryos with 50 μM SU5402 at the start of ampullary organ eruption (stage 39). In this case, compared with DMSO control embryos (n = 9; Fig. I,I′), no lateral line defects were observed in SU5402-treated embryos (n = 10/10; Fig. J,J′). We cannot rule out the possibility that treatment at higher dosages would have had an effect. Unfortunately, we were unable to repeat treatments under these conditions due to the limited spawning season in paddlefish, in which, at most, two clutches of embryos are available annually.

Taken together, these results suggest that two key stages, placode elongation and the start of ampullary primordia formation, are most important for Fgf-mediated regulation of hair cells and electroreceptors, respectively. However, at least to the extent to which we are able to assess this from these inhibitor experiments, the role(s) that Fgf plays in paddlefish lateral line organ development seem to differ significantly from what is observed during neuromast development in the zebrafish posterior lateral line, in which blocking Fgf signaling results in the loss of expression of the hair cell fate-determining transcription factor gene *Atoh1*, and the failure of epithelial rosette formation, i.e., the failure of hair cell specification and neuromast formation (Aman and Piotrowski, 2008, Lecaudey et al., 2008, Nechiporuk and Raible, 2008).

## 4 Discussion

The Notch and Fgf signaling pathways are both essential for neuromast formation and hair cell differentiation in the migrating zebrafish posterior lateral line primordium (reviewed by Aman and Piotrowski, 2011; Chitnis et al., 2012; Piotrowski and Baker, 2014; Thomas et al., 2015; Nogare and Chitnis, 2017). Here, we have provided the first reported evidence that these signaling pathways are also involved in neuromast and ampullary organ formation within sensory ridges formed by elongating (as opposed to migrating) lateral line placodes. Our results suggest conservation of the role of Notch signaling in preventing supernumerary sensory receptor cell formation in both neuromasts and ampullary organs. However, our data on *Fgf* ligand gene expression and the effects of blocking Fgf signaling suggest potential differences in the role(s) played by this pathway, both between different lateral organ types in paddlefish, and between paddlefish and zebrafish.

### 4.1 A potential role for *Jag1* in maintaining prosensory domains within the sensory ridges formed by elongating lateral line placodes

*Notch1*, the ligand gene *Jag1* and the presumed Notch effector gene *Hes5-like* were all expressed during neuromast and ampullary organ formation stages. Interestingly, *Jag1* expression was restricted to the developing lateral line system and otic vesicle, while *Notch1* and *Hes5-like* were expressed in many other structures, such as the brain and eye. This restricted expression pattern for *Jag1* suggests that this Notch ligand is likely only involved in the development of mechanosensory and electrosensory organs in paddlefish. *Jag1b* is also expressed in the developing lateral line system in zebrafish (Gwak et al., 2010), but its role there is unknown. In the zebrafish otic vesicle, *jag1b* is expressed in prosensory domains and maintained in sensory patches: it is required both for posterior crista survival (likely acting via Fgf10), and to prevent the spread of a region of Fgf-induced non-sensory cells that segregates the anterior and lateral cristae within the anterior prosensory domain (Ma and Zhang, 2015). In the mouse and chicken inner ear, Jag1-Notch signaling in Sox2-positive prosensory domains blocks cell differentiation, maintaining Sox2 expression and thus the competence of the prosensory domains to form both hair cells and supporting cells (Kiernan et al., 2006, Neves et al., 2011, Dvorakova et al., 2016), while lateral inhibition via Delta-Notch signaling, downstream of Atoh1 expression, determines which cells adopt a hair cell versus supporting cell fate (Neves et al., 2011). We speculate that Jag1 may play a similar role during lateral line organ development, maintaining prosensory domains within the sensory ridges formed by elongating lateral line placodes, which subsequently adopt neuromast versus ampullary organ fates. It is also possible that the spacing defects for both neuromasts and ampullary organs seen after blocking Notch signaling (see next section) relate to Jag1-Notch signaling being important for maintaining prosensory domains within the sensory ridge.

### 4.2 Persistent Notch signaling is required to prevent the formation of supernumerary lateral line hair cells and electroreceptors

We blocked Notch signaling pathway activity just before and during the emergence of each organ type, by applying the γ-secretase inhibitor DAPT for 18–24 h from stages 30–32 for neuromasts, versus from stage 36 for ampullary organs. This led to supernumerary hair cell and electroreceptor formation, and also to defects in the spacing apart of neuromasts and ampullary organs. Although no effect was seen on ampullary organ formation when DAPT was applied during placode elongation stages, the phenotypes reported above were observed for ampullary organs, as well as neuromasts, at all other stages tested, i.e., when the first ampullary organ primordia are forming, and even when ampullary organs are already erupting to the surface. Hence, persistent Notch signaling is required to stop supporting cells in both neuromasts and ampullary organs from adopting a sensory receptor cell fate. This is consistent with what is seen in zebrafish, where blocking Notch signaling led to the progressive expansion of *Atoh1* expression (Matsuda and Chitnis, 2010, Kozlovskaja-Gumbrienė et al., 2017).

However, there is also a significant difference between the overall effects of blocking Notch signaling in paddlefish versus zebrafish, since neuromasts (and ampullary organs) still formed in paddlefish, albeit with altered spacing (irregular spacing for neuromasts, with some clustering; clustering together for ampullary organs, in some cases leading to large patches of contiguous ampullary organs, which we speculate may relate to Jag1-Notch signaling being important for maintaining prosensory domains within sensory ridges; see previous section). In contrast, the progressive *Atoh1* expansion in the migrating posterior zebrafish lateral line primordium after blocking Notch is accompanied by the failure of epithelial rosette maturation, which is thought to result from attenuation of Fgf signaling (since Atoh1 downregulates *Fgfr1* expression, while Notch activity also downregulates the expression of Wnt genes, which normally activates Fgf ligand gene expression in the first protoneuromast; Matsuda and Chitnis, 2010; Kozlovskaja-Gumbrienė et al., 2017) and also from a direct requirement for Notch activity in supporting cell apical constriction and adhesion (Kozlovskaja-Gumbrienė et al., 2017).

### 4.3 Fgf inhibition results in fewer neuromasts but precocious ampullary organ development, with supernumerary receptor cells in both organ types

SU5402 treatment during paddlefish placode elongation stages, before or during the formation of the first neuromast primordia, led to the formation of fewer neuromasts, but with more hair cells, than in DMSO controls. (Treatment at this stage had no effect on ampullary organ formation.) SU5402 treatment at the stage when the first ampullary organ primordia are forming, similarly resulted in somewhat fewer neuromasts, though without obviously affecting hair cell number, but led to the precocious formation of ampullary organs with many more electroreceptors than in stage-matched DMSO controls. These contrasting results suggest that Fgf signaling may play different roles in neuromast versus ampullary organ formation in paddlefish.

Our data in paddlefish also contrast with the results of blocking Fgf signaling during zebrafish posterior lateral line placode development, which prevents *Atoh1* expression and epithelial rosette formation (i.e., prevents hair cell differentiation and neuromast formation) (Aman and Piotrowski, 2008, Lecaudey et al., 2008, Nechiporuk and Raible, 2008). In zebrafish, Fgf3 and Fgf10 activate Fgfr1 and hence *Atoh1* and Notch ligand expression in the central hair cell progenitor of protoneuromasts, with Atoh1 also inducing Notch ligand expression (Millimaki et al., 2007, Aman and Piotrowski, 2008, Lecaudey et al., 2008, Nechiporuk and Raible, 2008). However, it is not simply the case that SU5402 treatment at the stages examined here abrogates downstream lateral inhibition via Notch signaling within paddlefish lateral line organs, since DAPT treatment resulted in the formation of more neuromasts, rather than fewer, and did not accelerate ampullary organ formation.

Overall, our Fgf pathway inhibition data suggest there may be significant differences in the roles of Fgf signaling during sensory organ formation and receptor cell differentiation in elongating lateral line placodes, relative to what is known from the migrating posterior lateral line placode in zebrafish. However, a more detailed analysis of the timing of paddlefish lateral line organ formation and sensory cell specification is needed before any firm conclusions can be drawn in this regard.

### 4.4 Fgf ligand gene expression suggests multiple roles for different ligands

We found that *fgfr1* and the ligand genes *fgf3*, *fgf10* and *fgf20* seemed to be expressed at the same time during neuromast development in paddlefish; in contrast, developing ampullary organs lacked *fgf10* altogether, and showed significantly later expression of *fgf20* than either *fgfr1* or *fgf3*, suggesting that Fgf10 and Fgf20 likely play different roles in neuromast and ampullary organ development. In the mouse inner ear, genetic knockout studies have shown that Fgf10, acting via Fgfr2b - most likely redundantly with Fgf3 - is required for vestibular sensory patch formation (Pirvola et al., 2000, Pauley et al., 2003, Zelarayan et al., 2007, Urness et al., 2015). Fgfr1 is required for the formation of the organ of Corti (Pirvola et al., 2002), where Fgf20, most likely acting via Fgfr1, is required for the development of a subset of outer hair cells (Hayashi et al., 2008, Huh et al., 2012, Huh et al., 2015).

In mature zebrafish neuromasts at 4–5 days post-fertilization, *fgfr1* is expressed by supporting cells at the periphery of the neuromast (Steiner et al., 2014, Lee et al., 2016), while *fgf3* and *fgf10a* are restricted to hair cells, at the centre of the neuromast (Jiang et al., 2014, Lee et al., 2016). In mature lateral line organs at stage 46 in paddlefish (the onset of independent feeding), *fgfr1* was expressed more strongly at the periphery of neuromasts and within interneuromast cells; similarly, *fgfr1* was expressed at the periphery of ampullary organs and excluded from the central sensory epithelium. The ligand gene *fgf10* was expressed throughout the whole neuromast and in interneuromast cells, but was never expressed in developing ampullary organs. In contrast, *fgf3* and *fgf20* were expressed centrally in neuromasts, as was *fgf3* in smaller (presumably younger) ampullary organs. However, in larger (presumably mature) ampullary organs, *fgf3* showed weaker expression in the sensory epithelium and strong, patchy expression in a subset of peripheral cells; this pattern was also seen for *fgf20* in ampullary organs. The patchiness of this pattern suggests that in ampullary organs, *fgf3* and *fgf20* might be differentially expressed by a sub-population(s) of mantle cells (crescent-shaped cells that border the margins of both neuromasts and ampullary organs, e.g. Northcutt et al., 1994), as has been observed for a few genes in zebrafish neuromasts (Hernández et al., 2007, Steiner et al., 2014). This is interesting because mantle cells seem to be stem cells for new organ formation: in axolotl larvae, secondary neuromast or ampullary organ primordia form within the mantle layer of primary neuromast or ampullary organ primordia, respectively (Northcutt et al., 1994), while after tail/caudal fin amputation in both axolotl and zebrafish, mantle cells from the caudalmost neuromast on the stump act as stem cells for the formation of a migratory “regenerative placode”, which gives rise to neuromasts on the regenerating tail/caudal fin (Jones and Corwin, 1993, Dufourcq et al., 2006). We speculate that Fgf signaling in the mantle zone of mature neuromasts and ampullary organs could be involved in the maintenance of stem cells for secondary organ formation and/or organ regeneration.

### 4.5 Conclusions

This initial survey of the expression of specific Notch and Fgf signaling pathway genes during the development of elongating lateral line placodes on the head of a non-teleost ray-finned fish, and the effects of blocking these pathways at different stages of lateral line development using small-molecule inhibitors, suggest both conservation and possible divergence for the roles of these pathways in the formation of neuromasts versus ampullary organs, both within paddlefish, and between paddlefish and zebrafish. We hope that this work will stimulate further research to enable a more detailed understanding of the mechanisms underlying sense organ formation from elongating versus migrating lateral line placodes (or indeed versus sensory patch formation from prosensory domains in the inner ear). This will provide insight into how essentially identical lines of neuromasts over the head can be formed from a group of collectively migrating cells, versus a ridge of cells formed from an elongating primordium, and the extent to which these mechanisms are conserved with ampullary organ formation.

## Author contributions

M.S.M. and C.V.H.B. conceived the study and designed all experiments. M.S.M. performed all gene cloning and drug treatments, and most of the in situ hybridizations. O.R.A.T. performed some of the Fgf pathway gene in situ hybridizations and analyzed some SU5402 experiments. M.S.M. prepared all figures, with some contributions from O.R.A.T., except for Fig. 1, which was prepared by O.R.A.T. M.S.M. and C.V.H.B. wrote the manuscript. All authors read and commented on the manuscript.
